# Diagnostik und Therapie der Minimal Change Glomerulopathie beim Erwachsenen – 2023

**DOI:** 10.1007/s00508-023-02258-5

**Published:** 2023-09-20

**Authors:** Philipp Gauckler, Heinz Regele, Kathrin Eller, Marcus D. Säemann, Karl Lhotta, Emanuel Zitt, Irmgard Neumann, Michael Rudnicki, Balazs Odler, Andreas Kronbichler, Martin Windpessl

**Affiliations:** 1 Department Innere Medizin IV (Nephrologie und Hypertensiologie), Medizinische Universität Innsbruck, Innsbruck, Österreich; 2 Klinisches Institut für Pathologie, Medizinische Universität Wien, Wien, Österreich; 3 Klinische Abteilung für Nephrologie, Abteilung für Innere Medizin III (Nephrologie, Dialyse und Hypertensiologie), Medizinische Universität Graz, Graz, Österreich; 4 6. Medizinische Abteilung für Nephrologie & Dialyse, Klinik Ottakring, Wien, Österreich; 5 Medizinische Fakultät, Sigmund-FreudUniversität, Wien, Österreich; 6 Abteilung für Innere Medizin III (Nephrologie, Dialyse und Hypertensiologie), Akademisches Lehrkrankenhaus Feldkirch, Feldkirch, Österreich; 7 Vasculitis.at, Wien, Österreich; 8 Immunologiezentrum Zürich (IZZ), Zürich, Schweiz; 9 Department Innere Medizin IV (Nephrologie und Hypertensiologie), Medizinische Universität Innsbruck, Innsbruck, Österreich; 10 Department Innere Medizin 4 (Nephrologie und Hypertensiologie), Medizinische Universität Innsbruck, Innsbruck, Österreich; 11 Abteilung für Innere Medizin IV, Klinikum Wels-Grieskirchen, Wels, Österreich

**Keywords:** Minimal Change Glomerulopathie, Podozytopathie, Proteinurie, Glukokortikoide, Rituximab, Minimal Change Disease, Podocytopathy, Glucocorticoids, Rituximab

## Abstract

Die Minimal Change Glomerulopathie ist eine glomeruläre Erkrankung, die sich klinisch typischerweise als akut auftretendes nephrotisches Syndrom manifestiert. Die Diagnose wird bei fehlenden lichtmikroskopischen Veränderungen, jedoch typischem elektronenmikroskopischem Befund eines meist vollständigen Verlustes der podozytären Fußfortsätze mittels Nierenbiopsie gestellt. Das zumeist gute Ansprechen auf immunsuppressive Maßnahmen, insbesondere Glukokortikoide, lassen eine autoimmune Krankheitsgenese annehmen. Trotz allgemein guter Prognose können steroid-abhängige, steroid-resistente und häufig relapsierende Verläufe den Krankheitsverlauf komplizieren und den Einsatz alternativer Immunsuppressiva erforderlich machen. Die Österreichische Gesellschaft für Nephrologie (ÖGN) stellt hier einen gemeinsamen Konsens darüber vor, wie erwachsene PatientInnen mit Minimal Change Glomerulopathie am besten diagnostiziert und behandelt werden können.

## Abstract (en)

Minimal change disease is a glomerulopathy that clinically manifests as acute onset nephrotic syndrome. A diagnosis is made by renal biopsy, implying the absence of glomerular lesions on light microscopy but detection of extensive podocyte foot process effacement on electron miscroscopy. Considering the typically excellent response to immunosuppressive measures (especially to glucocorticoids), an autoimmune pathogenesis is assumed. Although general prognosis is overall beneficial, steroid-dependent, steroid-resistant and frequently-relapsing disease courses may complicate the management of these patients and necessitate the use of alternative immunosuppressive treatment strategies. Here, the Austrian Society of Nephrology (ÖGN) provides a consensus on how to best diagnose and manage adult patients with minimal change disease.

## Einleitung und Epidemiologie

Der traditionelle Begriff Minimal Change Glomerulopathie (Minimal Change Disease [MCD]) wurde für eine hochgradig proteinurische glomeruläre Erkrankung geprägt, die sich histologisch durch fehlende lichtmikroskopische Veränderungen auszeichnet, bei jedoch typischem elektronenmikroskopischem Befund eines meist vollständigen Verlustes der podozytären Fußfortsätze. Diese prominente Veränderung der Zellstruktur legt eine wesentliche Rolle der Podozyten bei der Pathogenese der Proteinurie nahe. Deshalb werden Erkrankungen mit derartigen histologischen Charakteristika (neben der MCD auch bestimmte Formen der fokal-segmentalen Glomerulosklerose [FSGS]) unter dem Begriff Podozytopathien zusammengefasst. Ihr exakter Mechanismus ist zwar meist unbekannt, wird jedoch als autoimmun vermittelt angenommen.

Im Folgenden werden MCD und FSGS getrennt voneinander betrachtet. Es sei jedoch auf die anhaltende Diskussion hingewiesen, ob es sich bei MCD und FSGS tatsächlich um zwei separate Krankheitsbilder handelt, oder diese vielmehr als verschiedene Manifestationsformen bzw. Krankheitsstadien einer gemeinsam zugrundeliegenden Erkrankung anzusehen sind [1]. In der Tat zeichnet sich in jüngster Zeit ein Paradigmenwechsel ab, bei dem sich Klassifikation und Behandlung von PatientInnen mit „primärer Podozytopathie“ nicht mehr rein anhand des histologischen Schädigungsmusters (MCD vs. FSGS), sondern vielmehr entsprechend der zugrundeliegenden Pathogenese, z. B. primär/idiopathisch/autoimmun vs. externe Noxe vs. genetisch ausrichtet [2, 3].

Die Podozytopathie in Form einer MCD ist die mit Abstand häufigste Ursache eines nephrotischen Syndroms im Kindesalter, liegt aber auch etwa 10–15 % aller Fälle eines primären nephrotischen Syndroms im Erwachsenenalter zugrunde [4]. Die MCD präsentiert sich klinisch als akut einsetzendes nephrotisches Syndrom, das meistens durch ein gutes Ansprechen auf Immunsuppressiva, insbesondere Glukokortikoide (GC), gekennzeichnet ist. Die Diagnose erfolgt durch eine Nierenbiopsie. Im Kindesalter wird aufgrund der guten Ansprechrate auf eine Therapie mit GC und der hohen diagnostischen Vortestwahrscheinlichkeit primär auf eine diagnostische Nierenbiopsie verzichtet und die Krankheit entsprechend als steroid-sensitives (SSNS) bzw. steroid-resistentes nephrotisches Syndrom (SRNS) bezeichnet.

Die MCD kann in jedem Alter auftreten, Männer sind häufiger betroffen als Frauen. Die Prognose ist gut und das Risiko einer terminalen Niereninsuffizienz gering. Trotzdem kann es im Krankheitsverlauf zu schwerwiegenden krankheits- oder therapiebedingten Komplikationen kommen. Komplizierte Verläufe umfassen steroid-abhängige (SA), steroid-resistente (SR) und häufig relapsierende (HR) Formen. Diese erfordern den Einsatz alternativer, steroidsparender Immunsuppressiva wie Calcineurin-Inhibitoren (CNI), Cyclophosphamid (CYC), Mycophenolsäure (MFS) oder B‑Zell-depletierender Substanzen wie Rituximab (RTX).

## Pathogenese

Der Entstehungsmechanismus der MCD ist nicht geklärt und vermutlich multifaktoriell. Virale Infektionen oder Allergene werden als mögliche Trigger angesehen. Eine Dysregulation bestimmter T‑Zell Subgruppen wird ebenso beschrieben [5, 6]. Für eine pathophysiologische Rolle von B‑Zellen in der Entstehung der MCD spricht der erfolgreiche Einsatz B‑Zell-depletierender Substanzen wie RTX [7]. Bislang nicht identifizierte „Permeabilitätsfaktoren“ führen schließlich zu einer Schädigung der Podozyten. In einer rezent veröffentlichten Untersuchung konnten erstmals anti-Nephrin Autoantikörper bei ca. 30 % der untersuchten MCD-PatientInnen nachgewiesen werden [8].

Die MCD tritt meist ohne erkennbaren Auslöser auf (primär). Insbesondere bei Erstmanifestation muss jedoch eine sorgfältige Evaluierung hinsichtlich sekundärer Ursachen erfolgen (siehe Tab. 1; [4, 9]). Die weiterführende Diagnostik erfolgt individuell.

**Table 1:** 

Kategorie	Beispiele (Auswahl)	Screening
Medikamente	Gold, Antibiotika (Ampicillin, Rifampicin), NSAR (diverse), Lithium, Metamizol, Tamoxifen, Interferon (β, µ), D‑Penicillamin, Probenecid, Sulfasalazin, Denosumab, Checkpoint-Inhibitoren, Etanercept	Anamnese
Infektionen	Respiratorische Infekte (bspw. viral), Syphilis, Tuberkulose, HIV, Mycoplasmen, Ehrlichiose, Borreliose, Echinokokkose, Schistosomiasis, div. Viren, Parasiten	Anamnese + Screening: HIV, Hepatitis, Tuberkulose; Weitere nach individueller Abwägung und Verdacht
Atopie/Allergie	Pollen, Hausstaub, Bienen, Lebensmittel (Kuhmilch, Ei)	Anamnese (Cave: IgE-Erhöhung auch ohne Atopie typisch bei MCD) [17]
Malignome	Hodgkin- & Non-Hodgkin Lymphome, Leukämie, Multiples Myelom, Thymom, Bronchial-Karzinom, Kolon-Karzinom, Kimura Syndrom (Angiolymphoide Hyperplasie mit Eosinophilie)	Anamnese + Klinische Untersuchung + altersentsprechende Vorsorgeuntersuchungen (Urologe/Gynäkologe, Dermatologe, Koloskopie, Röntgen-Thorax) + Serum-Elektrophorese + freie Leichtketten in Harn und Serum
Autoimmune Erkrankungen	SLE, Sjögren Syndrom, Diabetes mellitus Typ 1, Myasthenia gravis, Autoimmun-Pankreatitis, Zöliakie, Allogene Stammzelltransplantation	Anamnese + Screening auf ANA; Weitere nach ind. Abwägung
Vakzination	Influenza, Hepatitis B, Pneumokokken, Tetanus, Masern, Tollwut, Meningokokken, SARS-CoV 2	Anamnese

*NSAR* nichtsteroidale Antirheumatika, *HIV* humanes Immundefizienz Virus, *IgE* Immunglobulin E, *SLE* systemischer Lupus erythematodes, *ANA* antinukleäre Antikörper, *SARS-CoV‑2* schweres-akutes-Atemwegssyndrom-Coronavirus Typ 2

Im Rahmen der Basisdiagnostik sollten folgende Aspekte beachtet werden: Bei Erstmanifestation mit großer Proteinurie (z. B. Urinstix) sowie wenn die Einleitung oder Intensivierung einer immunsuppressiven Therapie erwogen wird, sollte das Ausmaß der Proteinurie mittels Bestimmung der Protein-Kreatinin und/oder Albumin-Kreatinin Ratio, idealerweise aus dem Morgenurin oder aus einem Aliquot des „versuchten Sammelintervalls“, bestimmt werden. Alternativ ist die Quantifizierung der Proteinurie im 24 h-Sammelurin möglich (Vorteil: präziser/Nachteile: aufwändiger, häufig Sammelfehler).

- Cave: Die Kreatinin-basierte Bestimmung der Nierenfunktion (sowohl Clearance als auch eGFR) ist nicht validiert für das nephrotische Syndrom. Aufgrund gesteigerter tubulärer Sekretion von Kreatinin kommt es beim nephrotischen Syndrom zur systematischen Überschätzung der GFR [18].
- Die Bestimmung von anti-Phospholipase A2 Rezeptor Antikörpern (anti-PLA2R Ak) mittels ELISA und/oder indirekter Immunfluoreszenztests (IIFT) sollte neben dem Ausschluss anderer potenzieller sekundärer Ursachen (siehe Tab. 1) bei jedem Erwachsenen bei Erstmanifestation eines nephrotischen Syndroms erfolgen.

Beim klinischen Bild eines akut einsetzenden nephrotischen Syndroms ohne direkt erkennbaren Auslöser (beispielsweise Nachweis von anti-PLA2R Ak) ist im Erwachsenenalter grundsätzlich die Indikation zur diagnostischen Nierenbiopsie gegeben.

## Diagnostik und Pathologie

Die Diagnose einer MCD kann ausschließlich histologisch gestellt werden. Das Fehlen lichtmikroskopischer Auffälligkeiten ist namensgebend für diese Entität. Auch die Immunofluoreszenz-Diagnostik ist üblicherweise unauffällig oder zeigt allenfalls eine milde mesangiale Akzentuierung und manchmal überwiegend diskrete IgM und/oder C1q Ablagerungen ohne elektronenmikroskopisches Korrelat. Dies scheint keine prognostische Relevanz zu haben und das Ansprechen auf die Therapie mit GC ist vergleichbar mit PatientInnen ohne Auffälligkeiten in der Immunofluoreszenz-Diagnostik [19].

Erst mittels Elektronenmikroskopie kann die charakteristische ausgeprägte Abflachung („*effacement*“) der Podozyten-Fußfortsätze nachgewiesen werden. Insbesondere bei älteren PatientInnen mit MCD können sich auch fokal interstitielle Fibrose und Tubulusatrophie im Sinne unspezifischer Alterungsprozesse finden. Weitere pathologische Befunde schließen automatisch die Diagnose MCD aus [4, 20].

## Klinik

Die MCD manifestiert üblicherweise abrupt (binnen Tagen bis wenigen Wochen) mit dem Vollbild eines nephrotischen Syndroms, definiert als Proteinurie ≥ 3,5 g/24 h bzw. ≥ 3 g/g Protein-Kreatinin Ratio begleitend von einer Hypalbuminämie (laborspezifische Grenzwerte) und Ödemen und einer Hyperlipidämie. Oft können die PatientInnen den genauen Zeitpunkt nennen, an dem die Ödeme erstmals auffielen. Die beträchtliche Wassereinlagerung geht häufig mit einer Gewichtszunahme einher. Bei älteren PatientInnen kommt es auch zu atypischen Manifestationsformen mit Hypertonie und eingeschränkter Nierenfunktion [1]. Eine Mikrohämaturie findet sich in bis zur Hälfte der Fälle (Tab. 2). Eine Krankheitsmanifestation kann auch mit einer akuten Nierenschädigung (AKI) vergesellschaftet sein (in Einzelfällen sogar mit Dialysepflicht) [21].

**Table 2:** 

	Häufigkeit	Risikofaktoren	Bedeutung/DD	Konsequenz
Arterielle Hypertonie	30 [22]–47 % [23]	–	–	–
Mikrohämaturie	28,9 [24]–58,5 % [25]	–	Häufige Begleiterscheinung; DD IgAN DD Alport Syndrom?	Weitere DD sind insb. bei Steroidresistenz zu erwägen
Moderat reduzierte GFR (ca. 25 % GFR-Reduktion)	Häufigkeit unterschätzt (Kreatinin-basierte eGFR überschätzt Nierenfunktion beim NS)	Schweres NS	Schädigungsmuster der Podozyten führt zur Fußfortsatzabflachung und -Verschmelzung; Permeabilität für Albumin erhöht, jedoch für kleinmolekulare Substanzen (Wasser, Harnstoff, Kreatinin, …) erniedrigt	Reversible, „benigne“ GFR-Reduktion um ca. 25 % (auch ohne Anstieg des Serum-Kreatinins möglich) [26]
Akute Nierenschädigung (> 1,5 × Serum-Kreatinin Anstieg)	10 [25]–40 % [21]	Männliches Geschlecht, Alter, Hypertonie, schwere Hypalbuminämie/Proteinurie, – Volumendepletion (diuretische Therapie) – Hochdosierte RASi – Nephrotoxische Komedikation	Mögliche DD: – MCD + IgAN – FSGS (tip lesion) – MCD + AIN – bilaterale Nierenvenenthrombose (potenzielle Hinweise: Schmerzen, LDH-Erhöhung, Makrohämaturie)	– RASi sollte (vorübergehend) pausiert werden – Passagere (über Tage bis Wochen) Dialysepflicht möglich (ca. 25 % der AKI Fälle) [26] – Risiko für anschließende CKD gegeben (aber gering) [24]
Thrombembolische Ereignisse	*Venös*: 24,1 % [27], 10–37 % [28]; *Arteriell*: 0,3 % pro Jahr [29]	Schwere Hypalbuminämie, weibliches Geschlecht, BMI ≥ 30, AKI, Sepsis, GC, Schwangerschaft [30]	–	Erwäge prophylaktische Antikoagulation (Vitamin‑K Antagonist/LMWH) nach Risiko-Nutzen Abwägung^a^ Cave: zu dOAKs existieren kaum Daten [31]

*DD* Differentialdiagnose, *IgAN* IgA Nephropathie, *GFR* glomeruläre Filtrationsrate, *NS* nephrotisches Syndrom, *MCD* Minimal change disease, *FSGS* fokal-segmentale Glomerulosklerose, *RASi* Inhibitoren des Renin-Angiotensin-Systems, *LDH* Laktatdehydrogenase, *AIN* akute interstitielle Nephritis, *BMI* Body-Mass-Index, *AKI* akute Nierenschädigung (acute kidney injury), *GC* Glukocortikoide, *LMWH* niedermolekulares Heparin (low-molecular-weight heparin), *dOAK* direkte orale Antikoagulantien

^a^Verweis: Separater Beitrag „Allgemeine Empfehlungen für die Behandlung glomerulärer Erkrankungen“

## Prognose

Die renale Langzeitprognose bei steroid-sensitiver MCD ist sehr gut und das Risiko einer terminalen Niereninsuffizienz äußerst gering [24]. Allerdings können insbesondere bei häufigen Rückfällen schwerwiegende Krankheits- oder therapieassoziierte Komplikationen wie Thromboembolien oder Infektionen auftreten.

Die MCD geht mit einer hohen Neigung zu Rezidiven einher, Rückfälle können noch Jahrzehnte nach Erstmanifestation auftreten. Obwohl die einzelnen Episoden zumeist gut mit GC beherrschbar sind, handelt es sich somit um eine potenziell chronisch-rezidivierende Erkrankung, worüber die PatientInnen bei Diagnosestellung aufgeklärt werden sollten. Spontanremissionen sind möglich, treten aber meist erst spät auf, sodass in Anbetracht der krankheitsassoziierten Komplikationen grundsätzlich eine immunsuppressive Therapie erfolgen sollte.

Bis zu 15 % aller Kinder und Jugendlichen mit SSNS haben auch als Erwachsene Rückfälle. Aus der relativ hohen Prävalenz einerseits und der Neigung zu Rezidiven andererseits ist eine Transition, also ein geplanter Übergang von der Kinderklinik in die Erwachsenenmedizin, kein unübliches Szenario. Derartige Situationen sollten nach Möglichkeit dazu genutzt werden, eine genaue Zusammenfassung des bisherigen Krankheitsverlaufs, kumulativer Immunsuppression, etwaiger manifester Toxizität (Minderwuchs …) zu erfassen beziehungsweise eine Basisdiagnostik (metabolisch, Knochenstoffwechsel) vorzunehmen, sowie individuelle Impflücken zu schließen.

## Therapie

Die Datenlage zur optimalen Behandlung der MCD im Erwachsenenalter ist limitiert. Derzeitige Therapiestrategien sind meist aus pädiatrischen Studien extrapoliert, beruhen auf retrospektiven Untersuchungen oder kleinen Fallserien [4].

Für den überwiegenden Teil der PatientInnen stellen GC die erste Wahl für die Initialbehandlung der MCD dar. Allerdings ist das Therapieansprechen im Vergleich zu Kindern und Jugendlichen verzögert und insgesamt weniger gut. Nur die Hälfte der PatientInnen erreicht nach 4 Wochen leitliniengerechter Behandlung eine komplette Remission (*complete remission* [CR]), die mediane Zeit bis zur Remission beträgt 2 Monate [4], in bis zu 25 % bleibt eine Remission überhaupt aus. Im Gegensatz zu anderen nephrotischen Glomerulopathien kommt es bei der MCD im Fall eines Therapieansprechens zu einer völligen Normalisierung des Harnbefundes, das heißt, ein Therapieansprechen folgt in der Regel einem „Alles-oder-Nichts“-Prinzip. Eine partielle Remission ist für die MCD untypisch und nicht selten Ausdruck einer anderen zugrundeliegenden Podozytopathie (z. B. „FSGS“), die in der initialen Diagnostik nicht erkannt wurde.

Das Ansprechen auf eine erste GC-Therapie erlaubt auch früh eine Prognoseeinschätzung, ein frühes Rezidiv ist bereits ein Prädiktor für einen ungünstigen Verlauf im Sinne von vermehrten Rezidiven.

Gemäß den KDIGO-Leitlinien soll die Erstmanifestation einer MCD wie folgt therapiert werden:

- Prednisolon in einer Dosierung von 1 mg/kg/d (Maximaldosis 80 mg/d). Die Einnahme sollte morgens zwischen 7 und 8 Uhr als Einzelgabe erfolgen. Das alternative alternierende Schema (Prednisolon 120 mg/d jeden 2. Tag) ist in Mitteleuropa ungebräuchlich. Aufgrund der ödembedingten passageren Gewichtszunahme sollte dabei das Körpergewicht vor Krankheitsmanifestation als Ausgangswert herangezogen werden.
- Nationale Guidelines und aktuelle kontrollierte Studien (TURING: *ISRCTN16948923*) limitieren die Höchstdosis auf 60 mg/d [32, 33].
- Die Mindestdauer dieser GC-Hochdosistherapie beträgt 4 Wochen (zu diesem Zeitpunkt erreicht die Hälfte der PatientInnen eine CR).
  Bleibt eine frühe CR aus, soll laut KDIGO die Anfangsdosierung für maximal 16 Wochen beibehalten werden. Über diesen Zeitraum erreichen dann etwa 80 % der PatientInnen eine CR.
  Wir sehen eine derartig lange Hochdosis-GC Therapie, die in Einzelfällen einer Gesamtdosis von bis zu 10 g Prednisolon-Äquivalent entspricht, insbesondere bei älteren PatientInnen kritisch und empfehlen bei ausbleibender Remission schon früher, beispielsweise nach 8 Wochen, alternative Behandlungsstrategien in Erwägung zu ziehen, obschon entsprechende Studien (RIFIREINS: *NCT03970577*) zum Zeitpunkt des Schreibens erst rekrutieren (siehe Alternativtherapien) [34].
- Nach Erreichen einer CR soll die GC-Therapie in unveränderter Dosierung noch für 2 weitere Wochen fortgeführt werden, bevor eine schrittweise Dosisreduktion („Tapering“) beginnen kann.
- Die optimale Dauer einer GC-Therapie ist unklar, auch fehlen einheitliche Empfehlungen für das Tapering, KDIGO empfiehlt eine Dosisreduktion von 5–10 mg Prednisolon-Äquivalent pro Woche, sobald eine CR erreicht ist [35].
- Die Gesamtdauer der Behandlung sollte längstens 24 Wochen betragen. Ein zu frühes oder rasches Ausschleichen birgt laut älteren Studien die Gefahr eines Rezidivs und der daraus resultierenden Problematik einer insgesamt höheren GC-Exposition [23].

**Figure 1:**
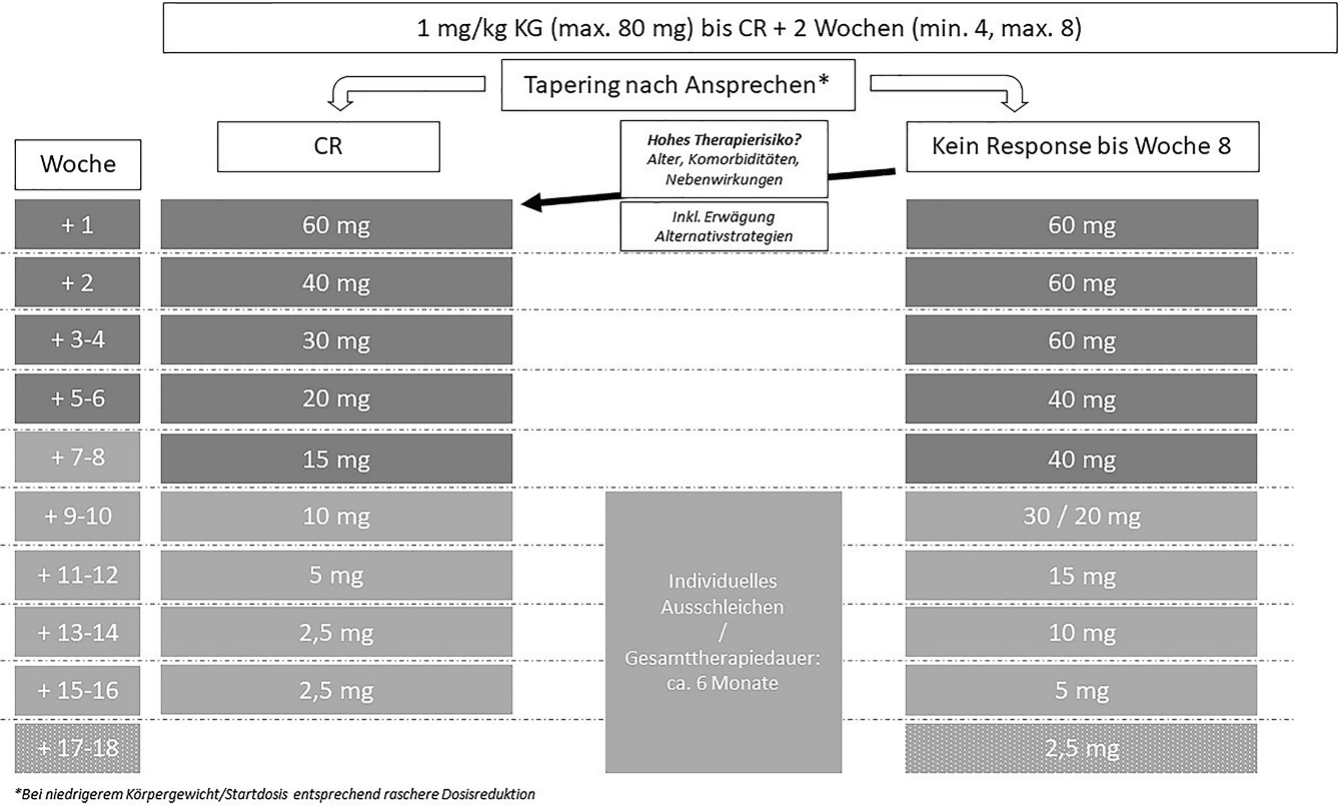

Ein Vorschlag für ein zeitgemäßes Niedrigdosis-GC Therapieschema ist in Abb. 1 dargestellt und orientiert sich am Protokoll der TURING-Studie [TURING: ISRCTN16948923]. Insbesondere der rechte Arm (kein Response bis Woche 8) weicht jedoch deutlich von KDIGO ab und beschränkt die Hochdosistherapie ab diesem Zeitpunkt auf maximal 60 mg/d Prednisolon für eine Dauer von höchstens weiteren 4 Wochen [4].

Die kumulative Gesamtdosis und die Therapiedauer unterschreiten die Empfehlungen aktueller Guidelines [20] deutlich und sind bis dato nicht durch Evidenz aus kontrollierten Studien gedeckt. Die Autoren sind dennoch der Meinung, dass ein solches Schema praktikabel, effizient und sicher ist.

Auch war ein rascheres Ausschleichen von GC in einer rezenten Publikation aus Japan effektiv und sicher. Rezidive traten bei raschem Ausschleichen zwar früher, jedoch nicht signifikant häufiger auf [33].

Bleibt eine CR auch nach 16 Wochen Therapiedauer (mit modifizierter Hochdosis GC-Therapie [s. oben] oder einer entsprechenden Alternativstrategie) aus, wird von einer Steroid‑/Therapieresistenz ausgegangen (SR-MCD). In derartigen Fällen sollte primär die Therapieadhärenz besprochen werden. Scheint diese gegeben, ist als nächstes die Diagnose zu reevaluieren. Dabei soll die Indikation zu einer Rebiopsie großzügig gestellt werden. Häufig findet sich dann eine FSGS, die entweder zuvor wegen eines *sampling errors* nicht erkannt wurde oder die sich erst im Krankheitsverlauf entwickelt hat. Grundsätzlich bedeutet Steroidresistenz eine schlechtere Prognose.

### Rezidiv (erneute Proteinurie > 3,5 g/d nach Erreichen einer CR)

Etwa zwei Drittel der erwachsenen PatientInnen, die primär eine CR erreichen, rezidivieren nach Beendigung der GC-Therapie, und selbst bei „Frühansprechern“ kommt es in gut 20 % der Fälle zu einem Rezidiv binnen eines Jahres [24]. Rund die Hälfte aller Rezidive tritt in den ersten 6 Monaten nach Beendigung der Steroidtherapie auf [24, 36]. Jüngere PatientInnen neigen eher zu Rezidiven als ältere.

Bei „seltenen Rezidiven“ (laut KDIGO-Definition < 4 pro Jahr) sollte die ursprünglich gewählte Therapie wiederholt werden, wobei im Fall einer GC-Therapie üblicherweise eine kürzere Therapiedauer ausreicht. Nach 4 Wochen oder Erreichen einer Remission wird die GC-Therapie in 5 mg-Schritten alle 3–5 Tage reduziert und nach 1–2 Monaten beendet. Bei häufigen Rezidiven (definiert als ≥ 2 Episoden binnen 6 Monaten oder ≥ 4 in 1 Jahr) sollte, um therapieassoziierte Nebenwirkungen einer prolongierten GC-Therapie zu vermeiden, eine alternative Behandlungstherapie erwogen werden (siehe unten).

Manchmal kommt es im Krankheitsverlauf zu selbstlimitierenden Rezidiven, die nur zufällig (z. B. wegen eines positiven Harnstreifens) auffallen. Bei asymptomatischen PatientInnen und stabiler Nierenfunktion kann dann für einige Tage das Eintreten einer Spontanremission abgewartet werden, bevor eine neuerliche immunsuppressive Therapie begonnen wird. Alternativ kann auch eine kurze, niedrig-dosierte GC-Therapie zielführend sein.

Grundsätzlich ist festzuhalten, dass die Definition von „seltenen Rezidiven“ in Einzelfällen eine beträchtliche kumulative GC-Exposition bedingen kann und bei derartigen, formal nicht „häufig-rezidivierenden“ Verläufen alternative Behandlungsstrategien geprüft werden sollten. Insbesondere RTX scheint uns hier eine gute Option (Details siehe unten). Aufgrund des potenziell chronisch-rezidivierenden Charakters der MCD empfiehlt es sich, die kumulative GC-Dosis prospektiv zu erfassen. Des Weiteren sollte der Therapiebeginn klar aus den Arztbriefen nachvollziehbar sein, um eine strukturierte ambulante Betreuung weiter zu gewährleisten.

### Alternativtherapien

**Figure 2:**
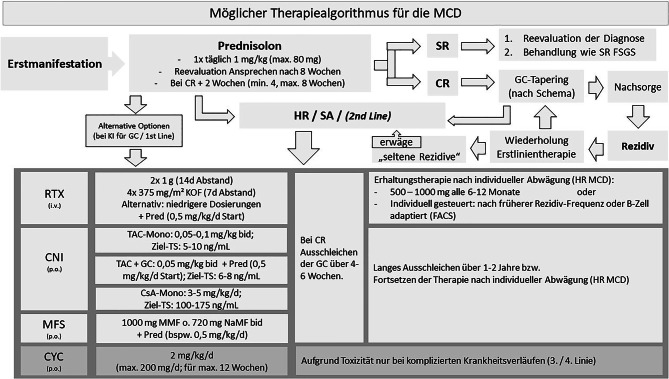

In Anbetracht der beträchtlichen Nebenwirkungen einer prolongierten hochdosierten GC-Therapie gewinnen daher, analog zum Management anderer autoimmuner Nierenerkrankungen, alternative Behandlungsansätze zunehmend an Bedeutung. Eine mögliche Vorgehensweise ist in Abb. 2 zusammengefasst.

Als „GC-sparende“ Therapiestrategien für die Erstlinientherapie der MCD kommen die beiden CNI Tacrolimus (TAC) oder Cyclosporin A (CsA), sowie RTX oder MFS in Frage, wobei uns insbesondere RTX als attraktive Option erscheint.

Die Auswahl erfolgt nach lokaler Erfahrung, Begleiterkrankungen sowie PatientInnenwunsch.

Mögliche Schemata sind:

- TAC-Monotherapie
  0,05–0,1 mg/kg bid (möglicher Talspiegel 5–10 ng/ml) [32, 37].
  In der MinTac-Studie wurde ein ursprünglich niedriger Talspiegel (4–8 ng/ml) erst bei ungenügendem Ansprechen nach 8 Wochen auf 9–12 ng/ml erhöht [32]. Dabei wurde eine CR unter TAC später erreicht als unter GC, erst nach 8 Wochen waren die Resultate vergleichbar. Bei verhältnismäßig kurzer Therapiedauer war die Rezidivhäufigkeit hoch [31]. Zur Therapiedauer s. unten
- Kombinationstherapie: TAC + niedrig dosierte GC
  0,05 mg/kg bid [Talspiegel 6–8 ng/ml] in Kombination mit niedrig dosierten GC (Startdosis: Pred 0,5 mg/kg/d) [38].
- CsA-Monotherapie
  3–5 mg/kg/d bid, Talspiegel: 100–175 ng/ml.

TAC zeichnet sich durch ein im Vergleich zu CsA günstigeres Nebenwirkungsprofil aus und wird deshalb heute allgemein bevorzugt. Beide Substanzen zeigen jedoch z. T. unterschiedliche Nebenwirkungen, sodass ein Wechsel angezeigt sein kann.

- Kombinationstherapie: GC + RTX
  Mögliche Therapieprotokolle in dieser Indikation werden im unteren Abschnitt (SA/HR MCD) besprochen.
- Kombinationstherapie: MFS + niedrig dosierte GC
  1000 mg (Mycophenolat-Mofetil) bzw. 720 mg (Natrium-Mycophenolat) bid, in Kombination mit niedrig dosierten GC (Prednisolon 0,5 mg/kg/d; maximal 40 mg/d). Bei Erreichen einer CR werden GC binnen 4–6 Wochen ausgeschlichen, MFS wird für eine Dauer von 24 Wochen fortgesetzt.

Aufgrund des beträchtlichen Nebenwirkungspotenzials sehen wir CYC nicht mehr als adäquate Erstlinientherapie bei GC-Kontraindikation an. Auch in der zweiten Linie liegen nun Daten für alternative Behandlungsansätze vor (zum Stellenwert von CYC bei HR/SA Verläufe s. unten).

Sowohl für CNI- als auch MFS-basierte Schemata gibt es keine klaren Empfehlungen bezüglich der Therapiedauer. Bei guter Verträglichkeit kann TAC über 1–2 Jahre eingenommen werden. Schleicht man die Therapie langsam aus, verringert sich das Rezidivrisiko im Vergleich zu abruptem Absetzen [39]. Man kann die TAC-Dosis nach einem Jahr in quartalsmäßigen Schritten um je 25 % reduzieren [34]. Das höhere Risiko eines Krankheitsrezidivs nach Beendigung der Therapie muss mit der Problematik der CNI-Nephrotoxizität bei langjähriger Einnahme abgewogen und mit dem PatientInnen diskutiert werden. Insbesondere bei PatientInnen mit vorbestehender Einschränkung der Nierenfunktion und/oder tubulointerstitieller Fibrose in der Biopsie sollte eine Behandlung mit CNI nicht zu lange fortgeführt werden.

### Steroid-abhängige (SA) *sowie* häufig-relapsierende (HR) MCD

In diesen Szenarien können gemäß der aktuellen KDIGO-Leitlinie orales CYC, CNI, RTX oder MFS als gleichwertige Optionen (vergleichbare Remissionsraten um 70–90 %) zum Einsatz kommen [20]. Die Entscheidung sollte nach sorgfältiger individueller Abwägung der jeweiligen Vor- und Nachteile (insbesondere Nebenwirkungsprofil), Verfügbarkeit und PatientInnen-Präferenz getroffen werden. Empfohlene Therapieschemata für CNI und MFS entsprechen den oben genannten. Wir sehen jedoch RTX inzwischen als geeignete, mit wenigen Nebenwirkungen behaftete, Option für die SA oder HR MCD an, zudem könnte sie bei möglicher Antikörper-mediierten Erkrankung als *targeted therapy* gesehen werden (s. oben). Üblicherweise geht einer RTX-Therapie ein GC-Wiederbeginn (in der Standarddosierung von 1 mg/kgKG) voraus.

Es gibt kein RTX-Standardschema und die Dosierung erfolgt üblicherweise nach den beiden bekannten Schemata (rheumatologisch/hämatologisch) entweder mit 2 × 1 g im Abstand von 2 Wochen oder 4 × 375 mg/m^2^ in wöchentlichen Abständen, wobei auch niedrigere Dosierungen verwendet werden (z. B. 375 mg/m^2^ im Abstand von 1 oder 2 Wochen). In einer Studie bei PatientInnen mit SA MCD war der Therapieeffekt besser (geringere Rezidivrate), wenn RTX nach Erreichen einer CR verabreicht wurde [40].

Wird eine anti-CD20-Therapie noch im manifesten nephrotischen Syndrom verabreicht, kann der renale Verlust des Wirkstoffs eine frühere neuerliche Gabe erforderlich machen. Aus diesen Gründen halten wir die RTX-Dosierung aus dem TURING-Protokoll (2 × 1 g im Abstand von 2 Wochen) für zweckmäßig (Details zu einer Therapie mit RTX: Siehe separater Beitrag „Allgemeine Empfehlungen für die Behandlung glomerulärer Erkrankungen“). Begleitende GC können dann binnen 1–2 Monaten oder noch rascher ausgeschlichen werden. In Einzelfällen, insbesondere bei HR-Krankheitsverlauf, wird zur Vermeidung neuerlicher Rezidive eine Erhaltungstherapie mit RTX etabliert (z. B. in halbjährlichen Abständen mit 500 mg). Diese Strategie wird zunehmend auch für PatientInnen mit formal „seltenen Rezidiven“ im langfristigen Verlauf gewählt [41].

Aufgrund der beträchtlichen Toxizität sollte eine Therapie mit CYC nur bei komplizierten Krankheitsverläufen durchgeführt werden. Zum Einsatz kommt orales CYC (2 mg/kg/d; 200 mg/d Maximaldosis) adjustiert für Alter und Nierenfunktion, über 12 Wochen. Diese Therapie resultiert in einer kumulativen CYC-Dosis 168 mg/kg (geschätzte Schwelle für gonadale Toxizität bei Männern: 200–250 mg/kg) [42].

- Eine CYC-Therapie sollte nicht länger als 3 Monate erfolgen und im Verlauf nicht neuerlich verabreicht werden. Die Toxizität der Behandlung muss mit den PatientInnen vorab ausführlich besprochen werden (Details zu einer Therapie mit CYC: Siehe separater Beitrag „Allgemeine Empfehlungen für die Behandlung glomerulärer Erkrankungen“).

### Steroidresistente (SR) MCD (ausbleibende Remission nach 16 Wochen Hochdosis GC Therapie)

Eine SR MCD tritt bei Erwachsenen in bis zu 10 % der Fälle auf. Wird eine korrekte Therapieadhärenz angenommen, muss die ursprüngliche Diagnose hinterfragt werden. Eine Rebiopsie sollte daher in Betracht gezogen werden, bestätigt jedoch zumeist entweder die Erstdiagnose (MCD) oder ergibt den Befund einer FSGS. Allerdings schließt auch der erneute Nachweis einer MCD eine zugrundeliegende FSGS nicht aus (sampling error). Die nachfolgende Therapie richtet sich zunächst in beiden Fällen nach den Empfehlungen der SR FSGS, sodass optional auch ein Therapieversuch mit einem CNI vor einer Rebiopsie erfolgen kann. Insbesondere bei jungen PatientInnen und/oder entsprechender Familienanamnese muss an dieser Stelle auch die Möglichkeit zugrundeliegender genetischer Ursachen und eine eventuelle genetische Abklärung berücksichtigt werden (Verweis auf den ÖGN-Konsensus: FSGS). Üblicherweise wird eine Behandlung mit einem CNI begonnen und parallel die laufende GC-Therapie reduziert/ausgeschlichen. Ein Therapieansprechen sollte nach einigen Monaten einer CNI-Behandlung ersichtlich sein. Eine begleitende niedrig dosierte GC-Therapie (5–7,5 mg/d) kann zusätzlich erfolgen. Im Fall eines Ansprechens sollte die Behandlung nach einem Jahr in Standarddosierung langsam ausgeschlichen werden. Der Stellenwert von RTX für die SR MCD ist bei Erwachsenen weniger gut belegt als bei Kindern. In einer kleinen Kohorte, die auch 5 PatientInnen mit Steroidresistenz beinhaltete, erreichten alle PatientInnen nach RTX-Gabe eine Remission [43].

### Supportivtherapie

- Grundsätzlich gelten die allgemeinen Empfehlungen für alle proteinurischen Glomerulopathien (siehe separater Beitrag „Allgemeine Empfehlungen für die Behandlung glomerulärer Erkrankungen“). Aufgrund der oft raschen Remission sind eine frühzeitige Behandlung mit RASi oder Statinen, sowie eine diätetische Eiweißrestriktion nicht zwingend erforderlich, sondern erst dann, wenn ein frühes Ansprechen ausbleibt.
- In ausgewählten Fällen ist eine Thromboseprophylaxe indiziert (siehe separater Beitrag „Allgemeine Empfehlungen für die Behandlung glomerulärer Erkrankungen“).
- Kochsalzrestriktion
- Diuretika („negative NaCl-Bilanz“)
- Blutdruckeinstellung
- Nikotinkarenz

Bereits zum Zeitpunkt der Erstdiagnose sollte vorausschauend ein Augenmerk auf mögliche zukünftige metabolische und infektiöse Nebenwirkungen der Immunsuppression gerichtet werden. Spätestens nach Erreichen einer Remission sollte in Abhängigkeit von anderen Risikofaktoren für eine Osteoporose (weibliches Geschlecht, höheres Alter, Rauchen …) eine Index-Densitometrie veranlasst werden (siehe separater Beitrag „Allgemeine Empfehlungen für die Behandlung glomerulärer Erkrankungen“).

In Remission sollte der Impfstatus aktualisiert und ein strukturiertes Impfprogramm diskutiert werden. Dieses beinhaltet neben jährlichen Influenza-Impfungen eine zweiteilige Pneumokokken-Impfung (PCV13 gefolgt von PPSV23) sowie eine Hepatitis‑B Grundimmunisierung. Obwohl in seltenen Fällen zeitliche Zusammenhänge zwischen Impfungen und *de-novo* MCD bzw. Krankheitsrezidiven berichtet wurde [16, 44], ist auch eine Schutzimpfung gegen COVID 19 bzw. Auffrischungsimpfungen nach erreichter klinischer Remission unbedingt zu empfehlen.

## Transplantation

Wegen des günstigen Verlaufs der Erkrankung sind Nierentransplantationen bei MCD eine Rarität und eher Ausdruck einer früheren Fehldiagnose, zumeist im Sinne einer initial „übersehenen“ FSGS oder einer anderen Zweitpathologie. Trotzdem kann eine MCD im Transplantat wieder auftreten [45]. Etwas häufiger ist das *de novo*-Auftreten einer MCD, wobei also kein Zusammenhang mit der Grundkrankheit besteht [46]. Die Manifestation erfolgt früh (fast immer binnen 4 Monate nach Transplantation); Lebendspenden dürften ein etwas höheres Risiko aufweisen. Fast immer wird durch GC eine CR erreicht.

## Schwangerschaft

*De novo* MCD in der Schwangerschaft ist äußerst selten. Therapieempfehlungen beruhen auf Fallberichten. GC, TAC und RTX wurden erfolgreich verwendet [47].

## Kontrollen

Über den oftmals chronisch-rezidivierenden Verlauf der Erkrankung sollen PatientInnen aufgeklärt werden. Selbstkontrollen mittels Urinteststreifen (beispielsweise im Rahmen eines Infekts oder nach Erreichen einer Remission in monatlichen Abständen) ermöglichen eine frühe Diagnose eines Rezidivs und einen raschen Therapiebeginn. Auch in anhaltender Remission erachten wir nephrologische Kontrollen, beispielsweise in jährlichen Abständen, für sinnvoll. In diesem Rahmen sollten auch Blutdruck und metabolische Parameter im Auge behalten werden, das Gewicht dokumentiert und der Impfstatus adressiert werden.
